# Climate change and mental health: a commentary

**DOI:** 10.1007/s44192-021-00001-y

**Published:** 2021-09-23

**Authors:** Julio Licinio, Ma-Li Wong

**Affiliations:** Precision Medicine Laboratory in Psychiatry (PMLP), SUNY Upstate Medical University, Institute for Human Performance, 505 Irving Ave 3302, Syracuse, NY 13203 USA

## Abstract

Climate change represents a major global challenge. Some hallmarks of climate change that have been connected to human activity include an increase of 0.8–1.2 °C in global temperatures as well as the warming of upper ocean water. Importantly, approximately 500 million people worldwide face the consequences of desertification. Simultaneously, the world population has grown from 1.6 billion in 1900 to 7.7 billion today, greatly exacerbating the human toll of devastating environmental disasters, which result in increasingly larger and more common mass migrations that also fuel human trafficking and modern-day slavery. The mental health outcomes are staggering and include, in the context of chronic stress, addiction, anxiety disorders, post-traumatic stress disorder (PTSD), bipolar disorder, major depression, and suicidality. Mental health practitioners, healthcare systems, and governments across the world need to be prepared to address the mental health sequelae of climate change.

The climate of our planet has always changed. However, that has happened in very extended geological epochs, while life exists in a much more abbreviated biological lifespan. What are the effects on mental health when the geological time frame of climate change is accelerated into our own biological time frame?

## Data on climate change: global warming and desertification

As we examine data on climate change, in order to ensure accuracy, we refer only to reports issued by the Intergovernmental Panel on Climate Change (IPCC), the United Nations body for assessing the science related to climate change. The IPCC stands out among the most reliable sources of scientific information on this key topic, and we would refer readers to their website, https://www.ipcc.ch, in order to gain free access to a wealth of data on this pressing subject. We will focus here on three of the IPCC multiple reports, namely those on global warming [1], the consequences of climate change on land use and humans [2], and the physical science basis demonstrating the heat content increase of the upper oceans (0–700 m) [3]. The IPCC estimates that “human activities are estimated to have caused approximately 1.0 °C of global warming above pre-industrial levels, with a likely range of 0.8–1.2 °C. Global warming is likely to reach 1.5 °C between 2030 and 2052 if it continues to increase at the current rate (high confidence)” [1]. The consequences of climate change on land, and consequently on humans, are summarized as follows:

*“About a quarter of the Earth's ice-free land area is subject to human-induced degradation (medium confidence). Soil erosion from agricultural fields is estimated to be currently 10 to 20 times (no-tillage) to more than 100 times (conventional tillage) higher than the soil formation rate (medium confidence). Climate change exacerbates land degradation, particularly in low-lying coastal areas, river deltas, drylands, and in permafrost areas (high confidence). Over the period 1961–2013, the annual area of drylands in drought has increased, on average by slightly more than 1% per year, with large inter-annual variability. In 2015, about 500 (380–620) million people lived within areas which experienced desertification between the 1980s and 2000s. The highest numbers of people affected are in South and East Asia, the circum Sahara region including North Africa, and the Middle East including the Arabian Peninsula (low confidence). Other dryland regions have also experienced desertification. People living in already degraded or desertified areas are increasingly negatively affected by climate change (high confidence)”* [2].

What stands out above is the extraordinary number of people (in the range of 500 million) living in areas that experience desertification. We are therefore facing the confluence of two major issues, namely climate change itself and the vast increases of the human population that make the impact of climate change much more substantial.

## Historical examples of devastating hurricanes

We often think of two natural disasters that happened before the current wave of climate change and wonder how devastating their effects would be today. We are referring to the Great Galveston hurricane of 1900 and the 1926 Miami hurricane.

The Great Galveston hurricane of 1900 and the accompanying tidal wave were the deadliest natural disaster in the United States history and the fifth-deadliest Atlantic hurricane overall. That category 4 hurricane left between 6000 and 12,000 fatalities in the United States; the number most cited in official reports is 8000. After viewing the destruction in Galveston, Clara Barton, founder of the American Red Cross, made the following statement:

*“It was one of those monstrosities of nature which defied exaggeration and fiendishly laughed at all tame attempts of words to picture the scene it had prepared. The churches, the great business houses, the elegant residences of the cultured and opulent, the modest little homes of laborers of a city of nearly forty thousand people; the center of foreign shipping and railroad traffic lay in splinters and debris piled twenty feet above the surface, and the crushed bodies, dead and dying, of nearly ten thousand of its citizens lay under them”* [4].

**Fig. 1:**
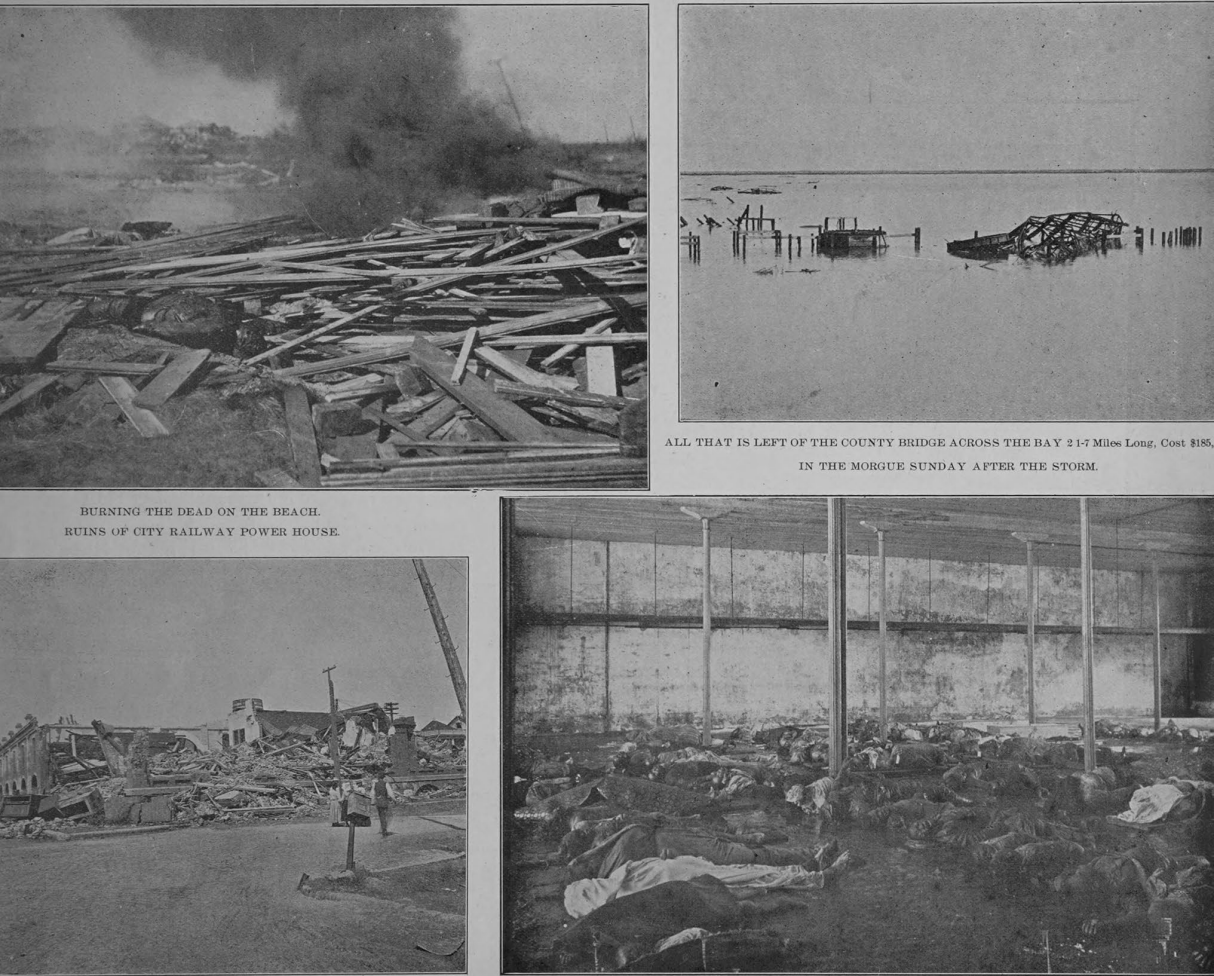
Top right-hand panel: Burning of the dead on the beach. Top left-hand panel: All that is left on the County Bridge across the bay 2 1–7 miles long. Bottom right-hand panel: Ruins of City Railway Power House. Bottom left-hand panel: In the morgue Sunday after the storm From ref. [5], in the public domain and free to use and reuse

**Fig. 2:**
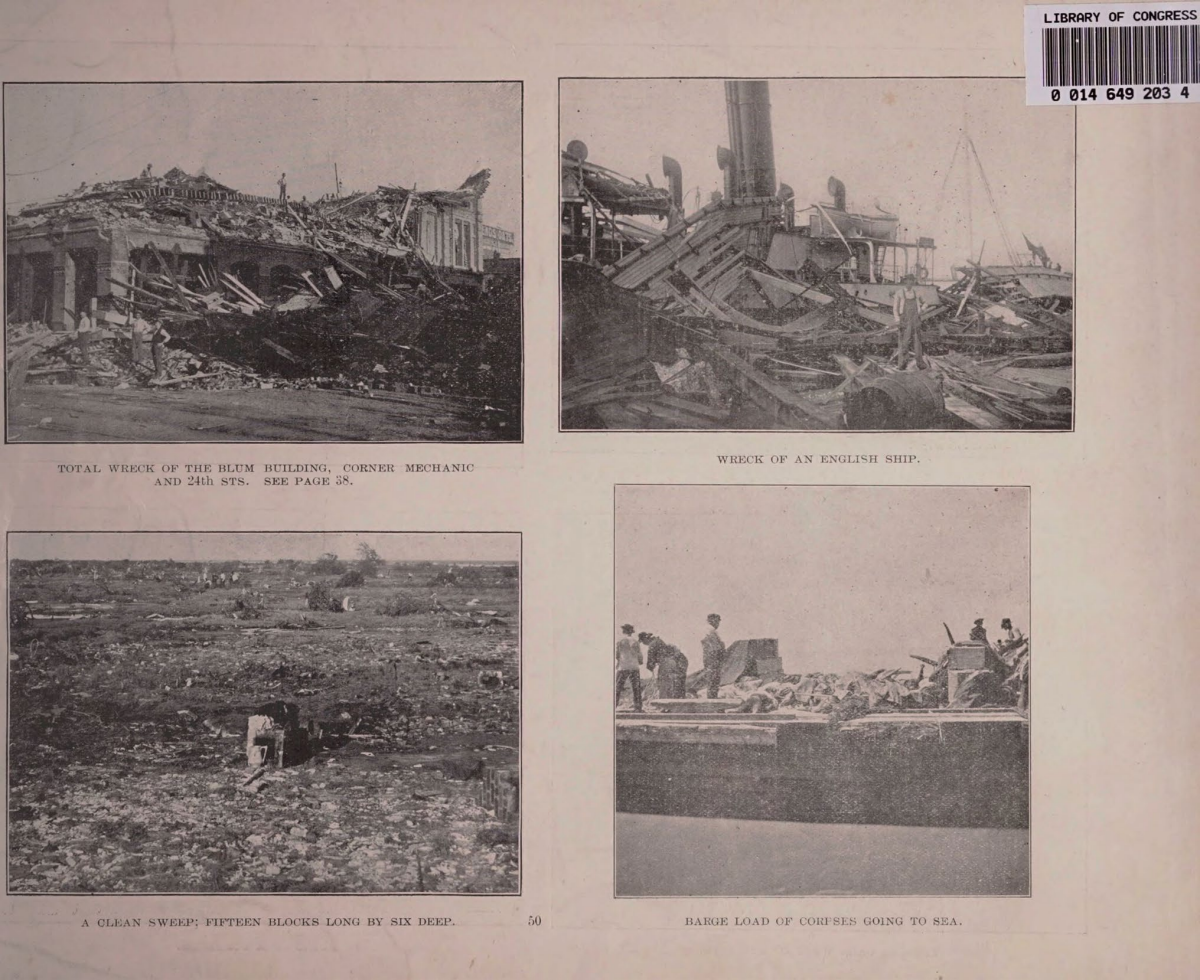
Top right-hand panel: Total wreck of the Blum Building. Corner Mechanic and 24th Sts. Top left-hand panel: Wreck of an English ship. Bottom right-hand panel: A clean sweep; fifteen blocks long by six deep. Bottom left-hand panel: Barge load of corpses going to sea. From ref. [5], in the public domain and free to use and reuse Credit: Library of Congress

Note that the number of residents for the metropolitan Houston area, which includes Galveston, was 182,000 in 1900. At that time, the entire state of Texas had 3.055 million residents. In contrast, by 2020, the Houston metro area had 7.154 million inhabitants, making it the fifth-largest metropolitan area in the U.S. The area’s population grew by an astounding rate of 3831% in 120 years, or 31.9% per year. The cataclysmic outcomes of the 1900 storm can be seen in Figs. 1 and 2 [5]. Can one even imagine what the outcomes of storms like that would be in an area that is now 3831% more inhabited than what it was back in 1900? The U.S. Department of Commerce’s National Oceanic and Atmospheric Administration states the following about hurricanes:

*“Hurricanes start simply with the evaporation of warm seawater, which pumps water into the lower atmosphere. This humid air is then dragged aloft when converging winds collide and turn upwards. At higher altitudes, water vapor starts to condense into clouds and rain, releasing heat that warms the surrounding air, causing it to rise as well. As the air far above the sea rushes upward, even more, warm moist air spirals in from along the surface to replace it. As long as the base of this weather system remains over warm water and its top is not sheared apart by high-altitude winds, it will strengthen and grow. More and more heat and water will be pumped into the air. The pressure at its core will drop further and further, sucking in wind at ever increasing speeds. Over several hours to days, the storm will intensify, finally reaching hurricane status when the winds that swirl around it reach sustained speeds of 74 miles per hour or more”* [6].

Not only has the IPCC concluded that human activity has led to a 0.8–1.2 °C increase in world temperature, but it has also stated the following:

*“It is virtually certain that upper ocean (0 to 700 m) heat content increased during the relatively well-sampled 40-year period from 1971 to 2010”* [3].

It is reasonable to think that if hurricanes occur over warm seawater, as seawater warms up globally, the likelihood of hurricanes being formed will likewise increase.

In 2006, we relocated to Miami, and at that time, we moved to an old house, built in 1924, whose claim to fame was the fact that it survived the catastrophic category 4 Great Miami Hurricane of 1926 that devastated the Greater Miami area. According to S. McIver, “the 1926 storm was described by the U.S. Weather Bureau in Miami as ‘probably the most destructive hurricane ever to strike the United States.’ It hit Fort Lauderdale, Dania, Hollywood, Hallandale, and Miami. The death toll is estimated to be from 325 to perhaps as many as 800. No storm in previous history had done as much property damage” [7]. Note that in 1926 Miami-Dade County had approximately 100,000 inhabitants; in 2021 that number was estimated to be 2,721,110. This represents a staggering population growth of 2621% in 95 years, or 25.6% per year—in a low-laying warm coastal location that is directly subjected to the effects of potentially calamitous hurricanes. The Great Miami Hurricane of 1926 was estimated to have caused damage of US$105 million in 1926 U.S. dollars. Weinkle and colleagues estimated that a similar storm would have caused economic losses of US$236 billion in 2017 [8].

## The double whammy of human population growth and climate change

Even without the impact of climate change, the global population growth represents by itself a major liability for the effects of climate on human activity. In 1900 there were 1.6 billion people on Earth; today there are 7.7 billion [9]. Pressing challenges for mankind include the outcomes of major climate events on increasingly large numbers of people as well as the fact that about 500 million people currently inhabit areas that are undergoing desertification. Because of the damage to our planet and its inhabitants caused by climate change, we could, within our lifetimes, be facing true tragedy on a scale that cannot yet be comprehended.

## Mental health outcomes of climate change

**Fig. 3:**
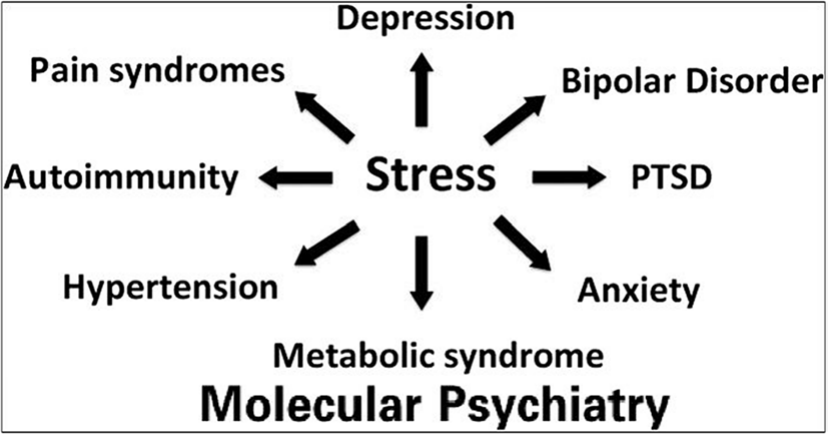
Stress as an example of pleiotropism in psychiatry. A single factor can be the cause of or contribute to a variety of diverse psychiatric and medical disorders. From ref [10], with permission

What would the effects of climate change-induced devastation be? The first effect is death, which could occur on a massive scale, depending on the extent and frequency of natural disasters. The consequences among survivors are myriad, going from physical disability caused by the direct effects of natural disasters to the sequelae of chronic stress. Chronic stress is pleiotropic, contributing to multiple physical and mental health outcomes, such as hypertension, metabolic syndrome, autoimmunity, pain syndromes, post-traumatic stress disorder (PTSD), addiction, bipolar disorder, major depression (including suicidality), and anxiety disorders, among others [10]—see Fig. 3. Chronic stress-related mental illness accelerates inflammation, which in turn exacerbates physical illness [11, 12]. The interactions of environmental factors, including chronic stress, with various types of genetic substrate, can also contribute to precipitate physical illness [13, 14].

Those who lose their homes or livelihoods as a result of catastrophic climate events and desertification may become refugees and migrate. Mass migrations, which are already occurring, represent a threefold blow to multiple countries worldwide: (1) The refugees’ native countries lose people who could contribute to their economy both as generators of economic activity as well as by being consumers. (2) The host countries face challenges absorbing and providing housing, education including language and cultural skills, employment, and healthcare to unexpected and vast numbers of people. (3) The migration process is sadly plagued by human traffickers who steer and place migrants into the underworlds of forced prostitution, drug trafficking, forced labor, and modern-day slavery. Human trafficking is a $150 billion industry globally [15]. We now have the largest number of slaves on Earth, more than we have had at any point in time throughout human history. In 2017, Alliance 8.7, a coalition of states and non-government organizations, estimated that there were some 40 million people enslaved worldwide, as well as 152 million child laborers [16]. Increases in climate change-related migrations will only exacerbate the crises of human slavery and forced child labor. It goes without saying that there are immense mental health consequences for the victims of human trafficking.

## Conclusions

Climate change represents a major challenge for mankind. Dramatic population growth in areas that are particularly vulnerable to climate change exacerbates the effects of climate change. The outcomes of natural disasters and desertification include mass migration which fuels human trafficking and its underworld of forced prostitution, drug trafficking, child labor, and slavery. There are already more slaves in the world today than at any other point in human history. Those numbers are likely to increase further as mass migrations accelerate. The impact on mental health includes, but is not limited to, addiction, anxiety disorders, post-traumatic stress disorder (PTSD), bipolar disorder, major depression, and suicidality. In the years ahead, addressing the exponential growth of mental health outcomes related to climate change will become an escalating challenge for mental health practitioners, healthcare systems, and governments worldwide.
